# On the role of the cellular prion protein in the uptake and signaling of pathological aggregates in neurodegenerative diseases

**DOI:** 10.1080/19336896.2020.1854034

**Published:** 2020-12-19

**Authors:** Giuseppe Legname, Carlo Scialò

**Affiliations:** Department of Neuroscience, Laboratory of Prion Biology, Scuola Internazionale Superiore Di Studi Avanzati (SISSA), Trieste, Italy

**Keywords:** Prion protein, oligomers, receptor, neurotoxicity

## Abstract

Neurodegenerative disorders are associated with intra- or extra-cellular deposition of aggregates of misfolded insoluble proteins. These deposits composed of tau, amyloid-β or α-synuclein spread from cell to cell, in a prion-like manner. Novel evidence suggests that the circulating soluble oligomeric species of these misfolded proteins could play a major role in pathology, while insoluble aggregates would represent their protective less toxic counterparts. Recent convincing data support the proposition that the cellular prion protein, PrP^C^, act as a toxicity-inducing receptor for amyloid-β oligomers. As a consequence, several studies focused their investigations to the role played by PrP^C^ in binding other protein aggregates, such as tau and α-synuclein, for its possible common role in mediating toxic signalling. The biological relevance of PrP^C^ as key ligand and potential mediator of toxicity for multiple proteinaceous aggregated species, prions or PrP^Sc^ included, could lead to relevant therapeutic implications. Here we describe the structure of PrP^C^ and the proposed interplay with its pathological counterpart PrP^Sc^ and then we recapitulate the most recent findings regarding the role of PrP^C^ in the interaction with aggregated forms of other neurodegeneration-associated proteins.

## 1. Introduction

Neurodegenerative disorders are characterized by the progressive failure of specific subsets of neurons associated with intra- or extra-cellular deposition of insoluble protein aggregates [1]. Extracellular deposits include aggregates with immunoreactivity for amyloid-β (Aβ) or prions (PrP^Sc^), while intracellular deposits include tau and α-synuclein (α-syn). Alzheimer’s disease (AD) is characterized by the progressive accumulation of extracellular pathological-associated plaques of Aβ and the presence of intracellular neurofibrillary tangles of hyperphosphorylated microtubule-associated protein tau [2,3]. In addition to AD, tau pathology or tauopathies include frontotemporal dementia linked to chromosome 17 (FTLD-17), progressive supranuclear palsy (PSP), argyrophilic grain disease (AGD), corticobasal degeneration (CBD) and Pick’s disease [4]. Lewy bodies (LB) and Lewy neurites (LN), characteristics of Parkinson’s disease (PD) and dementia with Lewy bodies (DLB), are mainly composed of aggregated α-syn, while oligodendroglial and neuronal α-syn deposits are present in multiple system atrophy (MSA) [5,6]. Prion diseases, or transmissible spongiform encephalopathies (TSEs), are unique infectious transmissible neurodegenerative disorder [7,8] with no clear evidence of naturally occurring human-to-human transmission of other neurodegenerative conditions [9,10]. Despite this distinction, clinical, cellular, molecular and biochemical studies provided evidence supporting a common mechanism of spreading and propagation of the neurodegenerative process [11–14]. The *prion-like* term is used to both indicate the similarities with the prion replication and propagation process and the lack of infectivity. The canonical model for the replication of misfolded protein lies in the prion paradigm [15]. In prion disorders, the conversion of the physiological normal cellular prion protein (PrP^C^) into its β-sheet enriched pathological conformer PrP-scrapie (PrP^Sc^) is central to the disease [16]. According to the *prion-like* hypothesis, misfolded protein assemblies in neurodegenerative diseases other than prion disorders, act as seeds of aggregation that can recruit their native isoforms and convert them into pathological molecules. The *seed* indicates the smallest amount of a misfolded protein which, once released in the extra-cellular space, is able to template and impose the pathological conversion onto native molecules and subsequently spread throughout connected brain areas [13]. Pathological investigations, genetic discoveries, animal and biophysics prediction models all supported a strict connection between pathologic protein aggregates and neurodegenerative diseases [17]. Emerging evidence has suggested that circulating soluble oligomers could play a role in the pathological process, while insoluble aggregates would represent protective less toxic counterparts [4,17–27]. Accordingly, the severity of cognitive deficits in AD correlates more strongly with the levels of soluble forms of Aβ than with insoluble amyloid plaque load [26] and impairment of hippocampal synaptic plasticity is detected before the formation of insoluble Aβ plaques in amyloid-β precursor protein (APP) transgenic mouse models of AD [28]. Moreover, soluble Aβ oligomers have shown detrimental effects on hippocampal long-term potentiation (LTP) *in vitro* and *in vivo* [26]. Similar effects have been shown for tau oligomers, either synthetic or extracted from AD diseased brains [21,29] and for *in vitro* obtained α-syn oligomers [30–32]. Aβ oligomers have been the most extensively investigated pathological species with multiple ligands identified [27]. Among these, PrP^C^ is the most prominent [33–38] and has been recently recognized as the highest affinity binding partner for Aβ oligomers [39]. Several studies have revealed that the binding between PrP^C^ and Aβ oligomers occurs at sub-nanomolar affinity interaction [19,34,35,37,40–46]. However, other studies have shown PrP-independent Aβ oligomer effects [47–50]. The hypothesis that PrP^C^, central in the pathogenesis of prion disorders, could represent a common acceptor for multiple neurodegeneration-associated protein species, has stimulated further investigations. As a consequence, in recent years the interplay between PrP^C^ and oligomers of proteins other than Aβ, such as tau and α-syn, has been increasingly investigated [19,22,23,51–55]. A possible interaction between tau and PrP^C^ was already suggested by neuropathological examinations of Gerstmann-Straüssler-Scheinker syndrome (GSS) cases, a subset of familial forms of TSEs. Mutant PrP assemblies display co-pathology with hyperphosphorylated forms of tau in GSS cases [56–63]. The nature of this co-pathology is still a matter of debate and further studies on the molecular interplay between PrP^C^ and tau could shed new light in the understanding of this phenomenon. Here we first describe the structure of PrP and the hypothesized interplay with its pathological counterpart, PrP^Sc,^ and then we will recapitulate the most relevant discoveries regarding the role of PrP^C^ in the interaction with aggregated forms of several neurodegeneration-associated proteins.

## 2. The prion protein structure and PrP^C^-mediated PrP^Sc^ toxicity

### 2.1 Prion protein structure

The cellular form of the prion protein, PrP^C,^ is a glycosylphosphatidylinositol (GPI)-anchored protein of 231 amino acids encoded in humans by the *PRNP* gene located on chromosome 20 [64–68]. The protein is structured in two regions: 1) an N-terminal flexible tail and 2) a globular C-terminal domain containing 3 α-helices and 2 short β-strands flanking the first α-helix. Recently, a third beta sheet strand has been described suggesting that the protein can adopt a more elaborate β0-β1-α1-β2-α2-α3 structural organization than the canonical β1-α1-β2-α2-α3 fold [69]. The flexible tail is further divided in a small charged cluster, an octarepeat (OR) region and a central domain, which comprises a second charged cluster and a hydrophobic domain (HD). The protein is translocated to the endoplasmic reticulum where it undergoes several post-translational modifications including N-linked glycosylation at residues N181 and N197, formation of a single disulphide bond at position C179 and C214, cleavage of the C-terminal signal peptide and subsequent attachment of the GPI anchor at position 231 [70]. PrP^C^ is widely expressed in the CNS during early development, in adult neurons and glial cells. Several putative functions have been suggested for PrP^C^, including ion balance homoeostasis, neuritogenesis, neuronal homoeostasis, cell signalling, cell adhesion and a protective role against stress [71]. PrP^C^ could serve as a dynamic platform for signalling modules at the cell surface, acting with the properties of a cell surface scaffold protein [72]. Since PrP^C^ does not span the plasma membrane, accessory molecules are required to transduce signals into the cytosol. Several PrP^C^ binding partners have been identified so far. The most studied are the metabotropic glutamate receptors (mGluR1 and mGluR5) *via* laminin γ1 chain interaction [73], the α7 type of nicotinic acetylcholine receptor (α7nAChR) following the binding of the cochaperone hop/STI1 [74], the neural cell adhesion molecule (NCAM) [75] and the Laminin Receptor Precursor/Laminin Receptor (LRP/LR) [76].

### 2.2 PrP^C^ is essential for prion replication

Several studies have shown that the expression of PrP^C^ is essential for prion propagation. Knock-out mouse models for PrP^C^ are resistant to prion diseases and to the propagation of the scrapie infectious agent [77]. In a seminal study, neural tissue overexpressing PrP^C^ was grafted into the brain of PrP-deficient mice, which were later inoculated with infectious prions. PrP^C^ deficient neurons, exposed to PrP^Sc^ material produced by PrP^C^ over-expressing neuronal grafts, did not show neuropathological alterations [78]. In another study it was shown that depleting endogenous neuronal PrP^C^ in mice with established prion infection reverted early spongiform changes and prevented neuronal loss and progression to clinical disease [79]. The expression of physiological levels of a form of PrP^C^ devoid of the GPI anchor (∆GPI-PrP) was permissive for PrP^Sc^ replication but produced a clinically silent phenotype [80]. When ∆GPI-PrP was expressed at higher levels it produced a late onset spontaneous phenotype associated with the deposition of large amyloid plaques of PrP^Sc^. Interestingly, disease onset was accelerated by co-expression of wild-type (WT) full-lenght PrP^C^ [81].

### 2.3 PrP^C^ mediates PrP^Sc^ toxicity

**Figure 1.**
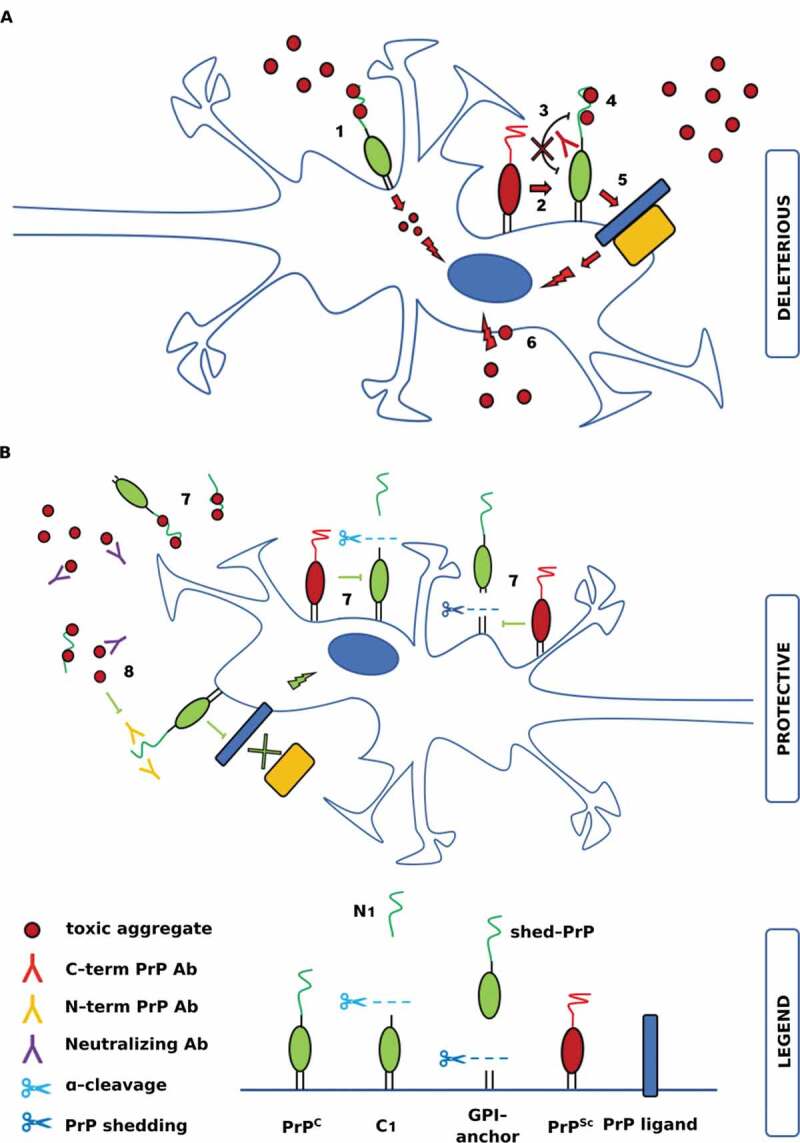
Schematic representation of the major features involved in the complex interplay between PrP^C^ and toxic pathological aggregates

All the afore-mentioned studies, in addition to confirming the role of PrP^C^ as an essential substrate for prion replication, indicated PrP^C^ as the cellular mediator of PrP^Sc^ neurotoxic effects. Moreover, different studies have suggested that PrP^C^ can acquire a neurotoxic role in the absence of PrP^Sc^/prion propagation [82,83]. A mouse model expressing a PrP mutant with a deletion of its hydrophobic domain (PrP∆HD) has shown that neurotoxicity of this deletion mutant was linked to a PrP^C^-dependent signalling pathway [83–85]. Importantly, these phenotypes were suppressed in a dose-dependent manner by co-expression of WT PrP^C^, suggesting that WT and mutated molecules could interact with each other, or compete for binding to a common molecular target that mediated both physiological and pathological effects. These toxic mutants of PrP^C^ induced spontaneous ionic currents, recordable by patch clamping techniques, when expressed in cell lines [86] and in primary neurons [87]. Notably, also these currents were silenced by co-expression of WT PrP^C^ in the same cells. Further studies of PrP∆HD mutants and their toxicity have suggested the presence of an auto-inhibitory mechanism that regulates the functional activity of PrP^C^, mediated by an intramolecular docking between N- and C-terminal domains of PrP^C^ [88]. According to this model, the toxic activity of the flexible N-terminal domain is regulated in *cis* by the globular structured C-terminal domain and anti-PrP antibodies directed against the C-terminal domain would produce a neurotoxic effect by disrupting this interaction. The authors of this study suggested that the same effect could be mediated by PrP pathological ligands, such as PrP^Sc^ or circulating pathological oligomers (Figure 1) [88]. Even if this model provides an interesting mechanistic explanation for oligomer-induced PrP^C^ mediated neurotoxicity, the fact that mice expressing N-terminally truncated PrP^C^ remained susceptible to prion diseases [89,90] argues against this being a primary pathogenic mechanism. Other studies have shown that PrP^Sc^ produced and released by ScN2a cronically infected cells induced apoptosis in SH-SY5Y cells, only when cells were transienly transfected to express PrP^C^ [82]. The scrapie prion-induced cell death was paralleled by the activation of the Jun N-terminal kinase (JNK) [82]. It should be stressed that the authors did not characterize whether the toxic PrP^Sc^ molecules were a PK-resistant intermediate generated during the conversion process or oligomers or fibrils. The same group repeated the experiment expressing heterologous PrP^C^ molecules, less suceptible to prion conversion due to species barrier. Heterologous PrP^C^, be it of hamster, human, cervid or bovine origin, efficiently mediated toxic signalling of mouse PrP^Sc^ [43]. Furthermore, they showed that PrP^Sc^ neurotoxic activity in SH-SY5Y cells expressing PrP^C^ can be reduced by the use of the oligomer-specific antibody A11 [43]. A similar PrP^C^-mediated neurotoxic effect was observed in the same experimental settings with Aβ oligomers [43].

Panel **A** represents all potential deleterious interactions between PrP^C^, PrP^Sc^ and toxic pathological aggregates. (**1**) PrP^C^ is involved in the uptake and internalization of tau and α-syn fibrils [51,52]. (**2**) The interaction between PrP^Sc^ and PrP^C^ is necessary for prion replication [77] and is also the driver of PrP^Sc^-induced toxic signalling [43], mediated by PrP^C^*via* several putative binding partners (**5**) [109]. (**3**) An auto-inhibitory mechanism regulates the functional activity of PrP^C^, mediated by an intra-molecular docking between N- and C-terminal domains of PrP^C^ [88]. Anti-PrP^C^ antibodies directed against PrP^C^ C-terminal domain (**3**) or PrP^C^ toxic ligands (**4**) produce a neurotoxic effect by disrupting this intra-molecular interaction. (**4**) PrP^C^ mediates Aβ, tau and α-syn aggregates neurotoxic effects *via* two binding sites located in its N-terminus [19,40,43,46] (**5**) PrP^C^ mediates cell signalling acting as an extracellular scaffolding protein, able to organize multiprotein complexes at the cell surface [72] (**6**) Toxic Aβ oligomers could bind directly to the cell membrane and disrupt its normal function, leading to cytotxicity [96]. N1 could bind to these species, or to the membrane surface, in a manner that prevents the formation of cell membrane damage (not shown). The **B** panel describes some of the hypothesized protective effects of PrP^C^ cleavage events and of anti-PrP^C^ antibodies. (**7**) PrP^C^ α-cleavage and shedding protective role: both the released N1 fragment and shed-PrP contain the N-terminal part of the protein harbouring binding sites for toxic aggregates, so, they could bind and neutralize them. The same effect could be induced by aggregate-directed neutralizing antibodies (in purple) [43]. Cleavage events decrease the presence of PrP^C^ at the cell membrane impeding the transmission of aggregate-induced toxic response(s). In prion disorders, cleavage events would reduce the amount of PrP^C^ which could be converted in PrP^Sc^ [92]. (**8**) Antibodies directed against the N-terminus of PrP^C^ showed a protective role against PrP^C^-mediated neurotoxic signalling, probably competing with toxic aggregates on their binding sites [22,23,35,110]

### 2.4 PrP^C^ proteolytic cleavage and its role in pathology

PrP^C^ is subject to post-translational proteolytic processing. These cleavage events have been shown to be involved in the regulation of its physiological functions, to produce biologically active fragments and to potentially influence pathology. Four cleavages appear to be conserved and currently represent the best investigated processing events on PrP^C^ [91,92]. The first proteolytic processing takes place in a region termed α-cleavage (located at positions K110/H111 or H111/M112 in the human sequence) and releases the N-terminal flexible part of PrP^C^ (N1 fragment of ~11 kDa) while leaving the globular C-terminal domain (C1 fragment of ~ 16 kDa) attached to the membrane *via* its GPI anchor [91–93]. Another minor proteolytic event termed β-cleavage occurs at residues 90–91. In consequence of this cleavage, an N-terminal fragment of ~ 9 kDa (termed N2) is released whereas a corresponding C2 fragment of 18–20 kDa (depending on the glycosylation status) remains bound to the cellular membrane. In contrast to α-cleavage, β-cleavage is mainly observed under pathological conditions and much less present or even absent in normal conditions. A third physiological cleavage of PrP^C^ occurs in close proximity to the GPI-anchor and results in the release of the almost full-length protein (shed-PrP) from the plasma membrane [91,92,94]. Recently, another proteolytic event of PrP^C^, *i.e*. γ-cleavage, has been characterized and described [95]. The exact cleavage site remains to be identified, but the molecular weights of the resulting fragments (i.e. a released N-terminal fragment N3 of ~ 20 kDa and a small GPI-anchored C3 fragment of ~ 5 kDa), suggest cleavage in a region between aa 170 and 200, possibly just N-terminal of the first N-glycosylation site. γ-cleavage seems to occur preferentially on unglycosylated PrP^C^ in the Golgi and trans-Golgi network as well as the endocytic recycling compartment and to be mediated by a metalloprotease [95]. Even if its pathophysiological roles are still unclear, the identification of the C3 fragment in Creutzfeldt–Jakob disease (CJD) brain samples suggests a possible link with pathology [92,95]. Several reports have highlighted the importance of α-cleavage to regulate PrP^C^ physiological functions [91,92], a detailed description of which goes beyond the scopes of this review. We will briefly discuss only those shown to have a potential influence in the interaction between PrP^C^ and pathological aggregates of proteinaceous species, PrP^Sc^ included. At least two modes of how proteolytic processing of PrP^C^ plays a role in disease have been suggested. First, both the released N1 fragment and shed-PrP contain the N-terminal part of the protein, which is thought to interact with pathological assemblies. Once released, they could bind and neutralize them. Second, cleavage events would decrease the presence of PrP^C^ at the cell membrane, thus impeding the transmission of aggregate-induced toxic response(s) as well as reducing the substrate for the conversion of PrP^C^ in PrP^Sc^, in prion disorders (Figure 1) [92]. Accordingly, the N1 fragment showed neuroprotective properties against PrP^Sc^ [43] and Aβ-mediated synaptic damage [34,96,97] and both PrP^C^ shedding and α-cleavage are increased during AD [98] (see next paragraph). Finally, since the N-terminal fragment is relevant for PrP^C^ internalization [99], its cleavage will result in an increased retention of the C1 fragment on the cell membrane. This has several beneficial effects. In fact, the C1 fragment cannot be converted in PrP^Sc^ and was also shown to act as a dominant negative inhibitor of PrP^C^ pathological conversion, further preventing PrP^Sc^ replication [100,101].

## 3. PrP^C^-mediated Aβ toxicity

### 3.1 Aβ oligomers and AD heterogeneity

Aβ pathological deposits encompass a mixture of peptides with different solubility, stability and biological properties [27], which can be present either in different areas of a single AD affected brain or in patients suffering from various subtypes of AD [102,103]. The heterogeneity in the length of Aβ peptides is related to different γ-secretase C-terminal cleavage positions in the amyloid precursor protein (APP). In fact, γ-secretase generates several peptides, of which Aβ43, Aβ42, Aβ40, Aβ38 and Aβ37 variants are the most abundant and studied (Aβ numbering indicates the last C-terminal amino-acid residue) [27]. Additional heterogeneity is generated by enzymatic processes mediated by aminopeptidases, glutaminylcyclases or isomerases, resulting in more than 20 Aβ peptides that participate in putative Aβ functions in the normal brain and oligomerization and fibrillization in the AD affected brain [27]. Aβ monomers do not interrupt normal synaptic function, whereas small Aβ oligomers and large Aβ aggregates (protofibrils) impair synaptic plasticity [26]. These different pathologic Aβ oligomeric circulating species likely contribute to the heterogeneity of AD clinical manifestations [104–107]. Aβ oligomers can be either obtained by *in vitro* aggregation of synthetic Aβ monomers or by collection from the culture medium of specific cell lines or from the soluble fraction of AD diseased brains [26].

### 3.2 PrP^C^ as Aβ oligomer receptor

Aβ oligomers have been shown to act as neurotoxins [24,26] with several putative ligands/receptors [27]. Among potential receptors, PrP^C^ has been extensively studied and has been recently recognized as the highest affinity binding partner for Aβ oligomers [39]. The first indication of PrP^C^ and Aβ oligomer interaction was obtained using an unbiased expression cloning screening approach for a specific class of Aβ42 synthetic oligomers, namely Aβ-derived diffusible ligands (ADDLs) [37]. ADDLs binding affinity to PrP^C^ was claimed in the nanomolar scale and the ADDLs-mediated blockade of LTP was absent in hippocampal slices from young adult PrP null mice. In addition, anti-PrP antibodies prevented ADDLs binding to PrP^C^ and were able to rescue synaptic plasticity in hippocampal slices. The deletion of 11 amino acids resulting in the Δ95–105 variant, significantly reduced this binding [37]. After this first finding, other studies confirmed PrP^C^-mediated Aβ oligomer neurotoxic effects [19,34,35,40–46,108-110] while others have shown Aβ oligomer deleterious effects which seem not to require PrP^C^ [47–50].

### 3.3 PrP^C^-mediated Aβ oligomer toxicity

Further investigations published also by authors who initially described PrP-independent Aβ toxicity, supported the role of PrP^C^ in mediating at least part of Aβ oligomer detrimental effects, suggesting that a consensus may be forming. Transgenic mice expressing increasing amounts of Aβ on a *PRNP* null background presented no detectable impairment of spatial learning and memory, despite displaying pathological levels of Aβ accumulation [108]. Basic amino acids 23–27 (KKRPK) at the very N-terminus of PrP^C^ were identified as a relevant Aβ oligomer binding site in addition to the previously characterized region encompassing residues 92–110 [40]. The toxic effect of *ex vivo* material extracted from human AD brain was absent in hippocampal slices devoid of PrP^C^. Furthermore, the anti-PrP antibodies ICSM-35 (which recognizes an epitope within the residues 93–102) and ICSM-18 (recognizing residues 143–153) were both able to block the Aβ-mediated inhibition of LTP *in vivo* and *in vitro* [35]. Co-cultures of SH-SY5Y cells expressing PrP^C^ with engineered CHO cells secreting toxic Aβ species in the culture medium, led to a significant increase in apoptotic cell death in SH-SY5Y cells transiently expressing PrP^C^, while control SH-SY5Y cells expressing GPI-anchored GFP displayed no adverse effects on cell viability [43]. Interestingly, SH-SY5Y cells expressing PrP^C^ did not exhibit increased apoptosis when co-cultured with CHO cells pre-treated with a γ-secretase inhibitor, showing that the detrimental effect was effectively driven by the presence of Aβ oligomeric toxic species in the culture medium. In the same study, they observed that toxic signalling via PrP^C^ required its intrinsically disordered N-terminal domain and the GPI anchor. Interestingly, a secreted version of PrP N-terminal domain was able to associate with Aβ conformers and to antagonize their toxic signalling [43]. Additional studies showed that soluble Aβ extracted from AD brains was able to bind to PrP^C^ at neuronal dendritic spines *in vivo* and *in vitro* where it formed a complex with fyn kinase, resulting in the activation of fyn activity. Furthermore, using the anti-PrP 6D11 antibody to prevent Aβ oligomer binding to PrP^C^, they abolished fyn activation and fyn-dependent downstream effects [110]. In the attempt to identify and characterize active PrP-dependent, PrP-independent and benign Aβ oligomeric assemblies, another study showed that Aβ assemblies that trigger PrP^C^-mediated toxicity are protofibrils with a defined three-dimensional structure [42]. The examined *in vitro* produced Aβ42 protofibrils presented an elaborated nanotube architecture with a triple helix structure wound around an hollow core [42].

### 3.4 PrP^C^ proteolytic cleavage and its role in AD pathology

Full-length PrP^C^ was able to inhibit *in vitro* Aβ40 and Aβ42 fibre formation and to cause disassembly of pre-formed Aβ mature fibrils, trapping Aβ in an oligomeric form enriched in antiparallel β-sheet [46]. Aβ recognition sites mapped to specific residues in the natively unstructured N-terminal half of PrP^C^, confirming the observations of previous studies pointing at residues 95–110 and 23–27 as primary and secondary binding enhancement regions. Accordingly, this interaction would maintain Aβ in a more toxic oligomeric conformation and thus promote pathology [46]. In contrast with these results, several studies have shown an *in vitro* and *in vivo* protective role for the released full-length recombinant PrP and its N-terminal fragment [34,45,96,97]. PrPN1, the main physiological cleavage fragment of PrP^C^, protected cultured primary neurons against toxicity and cell death triggered by oligomer-enriched conditioned medium collected from a CHO cell line overexpressing a mutated β*APP* gene [97]. PrPN1 also protected neurons against oligomers extracted from AD affected brain tissues [97]. The demonstration that PrPN1 was also able to bind early Aβ42 oligomeric intermediates during Aβ42 polymerization into amyloid fibrils [34] confirmed that PrP^C^ binding affinity is determined by positively charged residues in the two previously identified Aβ42 oligomer binding sites (positions 23–31 and 95–105). Interestingly, N1 strongly suppressed Aβ42 oligomer toxicity in cultured murine hippocampal neurons, in a *Caenorhabditis elegans*-based assay, and *in vivo* in a mouse model of Aβ-induced memory dysfunction [34]. Altogether these data suggest that PrP^C^ cleavage processing may play a relevant role in modulating AD pathology. This hypothesis was verified in the *APPswe/PSen1ΔE9* mouse model of AD, which showed altered PrP^C^ processing characterized by increased levels of both α- and β-cleavage in brain cortex [98]. Since the released N1 fragment contains the N-terminal part of the protein which is thought to interact with pathological oligomers, the observed increase in α-processing could be interpreted as a protective response with two potential beneficial functions: i) to decrease PrP^C^-mediated toxic signalling reducing the presence of PrP^C^ on the cell surface; ii) to neutralize Aβ oligomer pathological species before their binding to down-stream receptors [92]. On the other hand, the increase in β-cleavage, which has been previously associated to an excess of reactive oxygen species (ROS) [111], could reflect distinct processes such as lysosomal failure to clear Aβ aggregates and/or increased ROS production induced by stress, which is a common feature in AD mouse models [98]. Interestingly, other reports described a protective role against cytotoxic Aβ42 oligomers of soluble (membrane anchor-free) recombinant human prion protein (recHuPrP) and of the N1 fragment, also independently of the presence of endogenous PrP^C^ [96]. Both recHuPrP and N1 blocked Aβ42 oligomer mediated inhibition of LTP in hippocampal slices and in WT primary hippocampal neurons. Furthermore, they reduced Aβ42 oligomer-induced cytotoxic effects when the experiment was performed with hippocampal slices and neurons derived from *PRNP* null mice [96]. The authors explained these results suggesting a double protective action of recHuPrP and N1. The first mechanism would specifically target the PrP^C^-dependent LTP impairment, likely through a competitive inhibition of Aβ42 oligomer binding to PrP^C^ on the membrane surface. A second, less specific protective role against cytotoxicity, might be due to recHuPrP and N1 interference in the interaction between Aβ and the cell membrane: recHuPrP and N1 would bind to Aβ42 oligomer species, or to the membrane surface, in a manner that prevents the formation of defects and/or pores in the lipid bilayer of cell membrane [96]. These hypothesized mechanisms were supported by another study which analysed chemically cross-linked small oligomeric species of Aβ42, finding that recHuPrP inhibited the cytotoxicity of these species in neurons as well as their membrane permeation effects in liposomes [45]. Another recent study also supported a protective role of the N1 fragment and investigated the potential beneficial effect of monoclonal antibodies grafted with one of the two PrP-Aβ oligomer binding sites (i.e. aa 19–33 or 87–112) [41]. PrP-grafted antibodies bound a significant portion of aggregated Aβ in aqueous AD extracts, but when these antibodies were co-incubated with neurons treated with brain extracts, they did not reduce toxicity. By contrast, PrP fragment N1 did protect against Aβ toxicity [41]. Since conversely to N1, the two grafted antibodies harboured only one binding sequence each, this suggested that the presence of both Aβ oligomers binding sites on the molecule was a requisite for the PrP-mediated protective role [41].

### 3.5 PrP^C^ mediators of Aβ oligomer induced toxic signalling

It has been suggested that PrP^C^ may mediate cell signalling as an extracellular scaffolding protein, able to organize multiprotein complexes at the cell surface [72]. Several studies suggested that this scaffolding could also lead to neurotoxic effects. For instance, PrP^C^ binding to Aβ oligomers seems to produce a toxic signalling *via* metabotropic glutamate receptor 5 (mGluR5) [112], which in turn, initiates multiple changes in synaptic homoeostasis, leading to excitotoxicity, endoplasmic reticulum stress, and, eventually, to synaptic degradation and neuronal cell death [37,98,108,110,113–116]. Furthermore, PrP^C^ presence is enriched in post-synaptic densities (PSD) where the binding with *in vitro* and *ex vivo* derived Aβ oligomeric species activates the nonreceptor tyrosine kinase fyn, leading to NR2B phosphorylation and altered NMDAR localization and, ultimately, to destabilization of dendritic spines [44]. A mimicking peptide for the binding site of laminin γ1 to PrP^C^ (Ln-γ1), induced intracellular Ca^2+^ increase in neurons *via* the complex PrP^C^-mGluR5 [109]. Even if Ln-γ1 promoted the internalization of PrP^C^ and mGluR5 and transiently decreased Aβ oligomer binding to neurons, the peptide did not impact Aβ oligomer toxicity [109]. Since mGluR5 showed a key role in the transmission of toxic signalling also in prion diseases, the same group tested whether mGluR5 knock-out mice would be susceptible to prion infection, observing mild, but significant, effects on disease progression (i.e. delay in disease onset), without affecting survival of infected mice [109].

## 4. PrP^C^-mediated tau toxicity

### 4.1 Tau neurotoxic species

Tau is a microtuble-associated protein that under physiological conditions regulates microtubules assembly, dynamics and spatial organization [117]. The gene encoding tau, *MAPT*, is located on chromosome 17q21.3, spans approximately 150 kb and consists of 16 exons, only some of them constitutively translated [118]. Exons 2, 3 and 10 are subject to alternative splicing in the adult brain, giving rise to different tau isoforms that range from 37 to 46 kDa [119]. Interaction between tau and its partner, tubulin, is mediated by four imperfect repeat domains (encompassing 31–32 residues) encoded by exons 9–12 [120]. Alternative splicing of exon 10 results in the production of isoforms containing either 3 or 4 binding domains (3 R and 4 R tau) [121]. Tauopathies have as a common feature the accumulation of insoluble tau aggregates, called neurofibrillary tangles (NFTs), in the cell bodies of neurons and glia. Tau pathological aggregates differ in both phosphorylation and content of tau isoforms, which allows a molecular classification of tauopathies [4]. Recent evidence has implicated soluble, diffusible tau oligomers as important drivers of synaptotoxicity [21,29,117,122–124]. With the use of a tau oligomer-specific antibody, T22, it has been found that tau oligomers in human AD brain samples are four-fold higher than those in control brains and that tau oligomers are present in early stages of AD cytopathology [122]. *Post-mortem* analysis of brains from subjects affected by mild cognitive impairment have shown that cognitive decline was related with pre-fibrillar tau rather than NFTs [125,126] and several tau transgenic animal models have shown behavioural deficits, synaptic dysfunction, and cell death in the absence of NFTs formation [124]. Exogenous application of *in vitro* obtained soluble tau aggregates and tau oligomers extracted from AD brains impaired hippocampal LTP *in vitro*, and memory in mice [21,122]. Interestingly, tau oligomers could also act in combination with Aβ oligomers to produce these detrimental effects, since sub-toxic doses of the two species, administered in combination, led to LTP and memory impairment [21]. The same group observed that *APP* expression was required for an efficient intra-neuronal uptake of Aβ and tau oligomers [29]. Furthermore, *APP*-null mice were resistant to Aβ and tau oligomer induced impairment in memory and LTP, suggesting that the toxic effect was dependent upon *APP* expression [29].

### 4.2 PrP^C^-mediated tau oligomer toxicity

Since PrP^C^ has been identified as a receptor for PrP^Sc^ and Aβ oligomers (see above), recently many investigations have explored the interaction between PrP^C^ and other prion-like aggregated species. So far, few studies have shown a potential crosstalk between PrP^C^ and tau aggregates [19,23,52,53,127]. The possibility of an interaction between tau and PrP^C^ was already suggested by neuropathological examination of GSS cases which displayed the presence of co-pathology of PrP^Sc^ with hyperphosphorylated forms of tau [56–63]. Furthermore, full-length recombinant tau has been reported to bind to recombinant PrP^C^, *in vitro* [128]. In details, the N-terminus (amino acids 1–91) and tandem repeats region (amino acids 186–283) of tau were shown to be responsible for the interaction, which involved the octapeptide repeat domain on PrP^C^. The P102L GSS-related mutant and other PrP^C^ mutants characterized by the presence of two or seven extra octapeptide repeats (associated with familial forms of prion disorders) have shown enhanced tau binding affinity compared to WT PrP^C^ [128]. The first study showing a PrP^C^-mediated causality of tau oligomers, investigated the neurotoxic effect of secretomes of human induced pluripotent stem cell (iPSC)-derived models of AD [53]. In this study it was observed that the secretome of iPSC-derived neurons harbouring trisomy of chromosome 21 (Ts21secretome) was enriched in a complex mixture of extracellular tau species capable of inducing *in vivo* LTP impairment. Furthermore, they observed that this detrimental effect was prevented by tau immunodepletion of the Ts21 secretome or by pre-injection of the anti-PrP 6D11 antibody [53]. They presented comparable results using Aβ oligomer enriched secretomes of other iPSC-derived neuronal cell lines harbouring mutations in *APP* or *PS1* [53]. Intracerebroventricular injection of soluble aggregates formed from either WT or mutant P301S human recombinant tau potently inhibited hippocampal LTP *in vivo*, while tau monomers and end-stage fibrils appeared inactive [23]. Moreover, AD brain soluble extracts inhibited LTP in a tau-dependent manner. Their effect on LTP was abolished by tau immunodepletion or co-injection of a mid-region anti-tau monoclonal antibody, Tau5 [23]. Antibodies against the mid-region (6D11) and the N-terminus (MI-0131) of PrP^C^, also prevented the LTP inhibition induced by both recombinant and brain-derived tau, confirming a PrP^C^-mediated tau toxic effect [23]. Recently, another study provided evidence that soluble aggregates of tau bind to PrP^C^ *in vitro* and on mouse cortical neurons, *via* N-terminal PrP^C^ Aβ oligomer binding sites [19]. Tau soluble aggregates (produced *in vitro* or extracted from diseased brains) caused both functional (impairment of LTP) and structural (neuritic dystrophy) effects prevented by PrP^C^ ablation or pre-treatment with anti-PrP^C^ blocking antibodies [19]. In this study Corbett and colleagues proposed a standardized protocol to obtain soluble aggregates of tau (and also of Aβ and α-syn) which were isolated by progressive steps of centrifugation and sonication from *in vitro* obtained end-stage fibrils or by ultracentrifugation of brain homogenates and collection of their supernatant fractions [19].

### 4.3 PrP^C^ mediates tau uptake and complex tau-induced effects

Conversely to Aβ oligomers, *in vivo* injection of soluble full-length tau aggregates, increased the threshold for long-term depression (LTD) induction [127]. This effect was mediated by PrP^C^ and was reduced by systemic treatment with Ro 25–6981, a selective antagonist of GluN2B subunit-containing NMDA receptors [127]. Interestingly, the same molecule was not able to prevent tau-induced PrP^C^-mediated LTP. When co-administered with Aβ oligomers, tau soluble aggregates exhibited complex effects towards Aβ oligomer mediated LTD and LTP, completely blocking Aβ-facilitated LTD, while facilitating Aβ-induced LTP [127]. Taken together, these results suggested that both tau and Aβ oligomeric species interact with PrP^C^ to elicit opposite effects on synaptic plasticity, probably mediated by different effectors [127]. A complex interplay between PrP^C^ and tau fibrils has been recently shown. Fibrils of tau K18 (i.e. a truncated form of human tau containing only the 4 microtubule binding repeats) were actively internalized by N2a cells *via* PrP^C^ [52]. The uptake of tau K18 fibrils was reduced in PrP^C^-knockout N2a cells and by pre-treatment with antibodies against the N-terminus of PrP^C^. Surprisingly, exposure of ScN2a cells (i.e. chronically prion-infected N2a cells) to tau K18 fibrils, reduced PrP^Sc^ levels and increased PrP^C^ α-cleavage [52]. The study proposed that tau-induced clearance of PrP^Sc^ could be related to either an increase in PrP^C^ processing or to a direct binding of tau fibrils to PrP^C^, ultimately impeding its conversion in PrP^Sc^. These data suggest a bidirectional interaction between the two proteins [52].

## 5. PrP^C^-mediated α-synuclein toxicity

### 5.1 α-synuclein neurotoxic species

Synucleopathies are defined by the abnormal aggregation of α-syn with symptoms and clinical manifestations ranging from motor alterations to neuropsychiatric disturbances, cognitive decline and memory impairment [129]. LB pathology is not only confined to the *substantia nigra*, but extends to multiple brain areas including hippocampus and cortex, explaining the presence of non-motor symptoms [130,131]. Recent studies suggested that, as for other neurodegenerative diseases, α-syn oligomers may represent the most toxic α-syn species which, once released from neuronal cells, could contribute to the spread of pathology and, ultimately, to the major pathological features of α-synucleinopathies [32]. Extracellular α-syn oligomers, but not monomers or fibres, showed a detrimental effect on hippocampal LTP and increased basal synaptic transmission, through a mechanism dependent on NMDA receptor activation [30]. Furthermore, extracellular α-syn released from neuronal cells, was shown to bind to the Toll-like receptor 2 (TLR2), which, in turns, activated inflammatory responses in microglia, leading, eventually, to a neuroinflammatory response [31]. Interestingly, only specific types of α-syn oligomers were able to interact with and activate TLR2, suggesting that this α-syn-mediated TLR2 activation was conformation-sensitive [31]. Finally, Mao and colleagues suggested that the transmembrane protein, lymphocyte-activation gene 3 (LAG3/CD223) could be a neuronal receptor for synthetic α-synuclein preformed fibrils (PFF). Depletion of LAG3, or antibodies against LAG3, substantially reduced the transmission and consequent neurotoxicity of pathologic α-syn PFF [132]. In the last years, several studies described, sometimes with conflicting results [55], a number of α-syn toxic effects and mechanisms of inter-neuronal transmission, mediated, at least in part, by the cellular prion protein [19,22,51,133], which will be discussed in the next paragraph.

### 5.2 PrP^C^-mediated α-syn toxicity and uptake

Initial studies involving PrP^C^ in α-syn propagation and toxicity, compared the spreading and the extent of α-syn pathology in *Prnp*^+/+^, *Prnp*^0/0^ and Tga20 (i.e. overexpressing PrP^C^) mice injected, in the striatum, with α-syn PFF [133]. *Prnp* expression was not mandatory for α-syn transport in the mouse brain, although *Prnp*^+/+^ and Tga20 mice displayed increased levels of α-syn transport. Tga20 mice also showed increased Lewy body-like pathology in motor pyramidal neurons [133]. The same year, another independent study confirmed that PrP^C^ overexpression enhanced α-syn spreading and its pathologic deposition *in vitro* and *in vivo* [51]. Several studies observed α-syn binding sites on PrP^C^ [19,22,54] seemingly corresponding to the previously described PrP^C^ Aβ oligomer high affinity binding regions [40,46]. HEK293 cells expressing a PrP^C^ mutant devoid of its central domain (i.e. aa 90–110 or aa 91–115) presented significantly lower binding affinity for α-syn PFF [133] and α-syn oligomer mediated detrimental effect on LTP was abolished in hippocampal slices pre-treated with 6D11 anti-PrP antibody [22]. Very recently, another study has shown that Aβ, tau and α-syn protofibrils induced neurotoxic effects *via* PrP^C^ and their interaction was mediated by the two previously described Aβ oligomer binding site I (aa 23–33) and II (aa 88–111) [19]. Additional studies investigated putative effectors of α-syn oligomer-induced impairment of LTP and demonstrated that α-syn oligomers physically interacted with PrP^C^, inducing the phosphorylation of Fyn kinase *via* mGluR5 with the consequent activation of the NMDAR subunit 2B and altered calcium homoeostasis [22]. Notably, mGluR5 has been already described as a PrP^C^ binding partner in the transmission of Aβ oligomer-induced toxic signalling [109,112]. In contrast, another study, found that PrP^C^ was not mandatory to mediate α-syn oligomer detrimental effects *in vitro* and *in vivo* [55]. α-syn oligomer toxicity was comparable in *Prnp*^+/+^ and *Prnp*^0/0^ neurons and both *Prnp*^+/+^ and *Prnp*^0/0^ mice injected with α-syn oligomers presented memory deficits and hippocampal gliosis. Furthermore, surface plasmon resonance experiments showed no interaction between PrP^C^ and the preparation employed of α-syn oligomers [55]. These latter results suggest that further experiments are needed to address the relationship between PrP^C^ and α-syn pathological species. As for Aβ oligomers, it is possible that different pathological conformers influence the binding with PrP^C^ and drive PrP^C^-independent toxic effects. To clarify this point and to evaluate the significance of PrP^C^ in α-synucleinopathies, it is of utmost importance to extend the characterization and to standardize the preparation protocols of tested aggregates, as recent studies have pointed out [19]. As for tau, also α-syn fibrils presented a surprising effect on prion clearance in an *in vitro* cellular system [51]. When fibrils of α-syn were added to N2a cells chronically infected with prions, they induced a reduction in the levels of the PK-resistant PrP^Sc^ material and an increase in PrP^C^ α-cleavage processing, confirming that the interplay between pathological proteinaceous aggregates and PrP^C^ could involve bidirectional effects [51,52].

## 6. Conclusions and future perspectives

The interaction between PrP^C^ and Aβ toxic oligomeric species seems now widely accepted. As a consequence, several studies have extended their focus to validate the hypothesis that PrP^C^ mediates the uptake and the toxic signalling induced by aggregates of proteins other than Aβ, such as tau and α-synuclein. PrP^C^ interaction with these toxic species seemingly involves complex mechanisms of signal transduction and is likely influenced by PrP^C^ physio-pathological cleavage processing. Moreover, initial reports suggested that the interplay between PrP^C^ and pathological aggregates could lead to bidirectional effects. Figure 1 schematically illustrates the major proposed PrP^C^-mediated effects discussed in this review. Lessons learned from the first studies on PrP^C^ and Aβ interaction could be used as a guide to design future investigations and to correctly interpret resulting data. First of all, it is now clear that the preparation protocol used to obtain synthetic aggregates is a key element to increase the reproducibility of results and to help in the characterization of the putative pathological conformer responsible for PrP^C^-mediated toxic effects. Related to this point, patient-derived Aβ, tau and α-syn enriched samples are extremely precious, and it would be reasonable to collect data on *in vitro* obtained aggregates and to subsequently verify their biological relevance as a second step. A second point is that studies should report both positive and negative results, which should help to understand the mechanistic processes involved in the transmission of toxicity. Faster understanding of the biological significance and central role of PrP^C^ for many neurodegenerative disorders may have relevant therapeutic implications. If the nature and extent of the interaction between PrP^C^ and neurodegenerative pathological aggregates will be confirmed, PrP^C^ could be the target of several therapeutic approaches, as for instance the use of antisense oligonucleotides to knock-down the *PRNP* gene *via* RNA interference, the administration of PrP^C^ derivatives (i.e. the full-length recombinant protein or its N-terminal fragment) or anti-PrP^C^ antibodies. In this regard it should be mentioned that the use of anti-PrP^C^ antibodies in therapeutic applications might be limited to avoid undesired detrimental effects due to the disruption of physiologically relevant and protective PrP^C^ interactions.
